# TLR4 Deficiency Exacerbates Biliary Injuries and Peribiliary Fibrosis Caused by *Clonorchis sinensis* in a Resistant Mouse Strain

**DOI:** 10.3389/fcimb.2020.526997

**Published:** 2021-01-05

**Authors:** Chao Yan, Jing Wu, Na Xu, Jing Li, Qian-Yang Zhou, Hui-Min Yang, Xiao-Dan Cheng, Ji-Xin Liu, Xin Dong, Stephane Koda, Bei-Bei Zhang, Qian Yu, Jia-Xu Chen, Ren-Xian Tang, Kui-Yang Zheng

**Affiliations:** ^1^Jiangsu Key Laboratory of Immunity and Metabolism, Laboratory of Infection and Immunity, Department of Pathogenic Biology and Immunology, Xuzhou Medical University, Xuzhou, China; ^2^National Experimental Demonstration Center for Basic Medicine Education, Department of Clinical Medicine, Xuzhou Medical University, Xuzhou, China; ^3^Huai‘an Center for Disease Control and Prevention, Huai‘an, China; ^4^National Institute of Parasitic Diseases, Chinese Center for Disease Control and Prevention, Key Laboratory of Parasite and Vector Biology, Ministry of Health, WHO Collaborating Center of Malaria, Schistosomiasis and Filariasis, Shanghai, China

**Keywords:** TLR4, *Clonorchis sinensis*, cholangiocytes, fibrosis, C57BL/10 mice

## Abstract

Mice with different genetic backgrounds have various susceptibilities to infection with *Clonorchis sinensis*, although the mechanisms underlying are largely unknown. Toll-like receptor 4 (TLR4) as one of the most important pattern recognition receptors (PPRs) is essential for the invasion, survival, pathogenesis, and elimination of worms. The roles played by TLR4 in *C. sinensis* infection may vary due to the different genetic backgrounds of mice. In the present study, a relatively resistant mouse strain-C57BL/10 to *C. sinensis* was used for investigation on the possible roles of TLR4 in the biliary injuries and peribiliary fibrosis. TLR4 wild type (TLR4*^wild^*) and TLR4 defective (TLR4*^def^*) mice were orally infected with 45 metacercariae of *C. sinensis*, and all *C. sinensis*-infected mice and non-infected groups were anesthetized on day 28 post-infection. The liver and serum from each mouse were collected for assessment of the biliary injuries and biliary fibrosis. Meanwhile, hepatic leukocytes were isolated and detected for the activation of M1 or M2 macrophage using flow cytometry. The hepatic type 1 immune response and type 2 immune responses -relative molecules were also evaluated using ELISA and quantitative PCR. The data showed that TLR4*^def^* aggravated liver inflammatory cell infiltrations, bile duct proliferation, biliary and hepatocellular injuries, and ECM deposition in *C. sinensis*-infected mice, compared with TLR4*^wild^* mice when they were intragastrically administered with the same amounts of *C. sinensis* metacercaria. Furthermore, the M2-like macrophages and type 2 immune responses were significantly predominant induced in TLR4^*def*^ mice, compared with that of TLR4*^wild^* mice following *C. sinensis* infection. But the type 1 immune response were significantly decreased in TLR4*^def^* mice, compared with TLR4*^wild^* mice after *C. sinensis* infection. These data demonstrate that TLR4 deficiency exacerbates biliary injuries and peribiliary fibrosis caused by *C. sinensis* in C57BL/10 strain mice, which is contributed by augments of type 2 immune responses and decrease pro-inflammatory responses.

## Introduction

*Clonorchis sinensis* is a zoonotic food-borne parasite which infects human or other mammals *via* ingestion of raw or undercooked fresh fish and shrimp containing metacercaria (Tang et al., 2016). The adult worm mainly dwells in the intrahepatic bile duct, common bile duct, or gall bladder, although it can be also accidentally found in the pancreatic duct (Kim, 1999). Infection with *C. sinensis* caused 5,591 deaths every year and 275,370 disability-adjusted Life Year (DALYs) (Sripa, 2012), of which ranges from cholangitis, obstructive jaundice, gallstones to hepatic fibrosis due to its mechanical stimulation and production and secretion antigen (Hong and Fang, 2012; Zheng et al., 2015). In addition, a long-term infection by *C. sinensis* can potently induce bile duct carcinoma and *C. sinensis* is considered as a type I Biological carcinogen (Tyson and El-Serag, 2011; Zheng et al., 2015).

Toll-like receptors (TLRs) are an important family of pattern recognition receptors (PRRs), which play a very important role in the innate immune response as well as adaptive immune responses (Akira et al., 2001). It is widely expressed in immune cells (such as macrophages, dendritic cells, NK cells, lymphocytes, granulocytes) and non-immune cells (such as epithelial cells, endothelial cells, fibroblasts, cancer cells, etc.), and is involved in almost all human disease processes (Ciferska et al., 2020). Among the 13 TLRs in human or 11 TLRs in mice, TLR4 has been the first to be identified and has a relatively broad ligand specificity. TLR4 can interact with LPS, peptidoglycan, glycoprotein, and so on, and ultimately produce pro-inflammatory cytokines and inflammatory chemokines through MyD88 or TRIF adaptor protein to regulate immunity, which play critical roles in fighting against pathogenic insults (Iwasaki and Medzhitov, 2004). For example, upon binding to the ligand, TLR4 can activate nuclear transcription factors NF-κB or MAPK to induce the release of pro-inflammatory cytokines and regulate activation of macrophage by MyD88 dependent signaling pathway and TRIF-dependent signaling pathway (Li and Cherayil, 2003; Jiang et al., 2015; Molteni et al., 2016). However, recent studies also showed that the TLR4-induced signaling pathway participates in the homeostasis of the tissue and orchestrated tissues repair after damages caused by various insults. Studies have confirmed that TLR4 plays an important role in liver injury (Cengiz et al., 2015; Seki and Schwabe, 2015; Gandhi, 2020).

Our previous study also showed that TLR4 promoted peribiliary fibrosis by orchestrating the TGF-β signaling pathway in a susceptible mouse model (C3H/HeN mice) (Yan et al., 2017). However, the roles of TLR4 might vary in the different genetic backgrounds of the mouse following the encounter with different stimuli. In the present study, we used TLR4*^wild^* (C57BL/10JNju, TLR4*^wild^*) and TLR4*^def^* (C57BL/10ScN, TLR4*^def^*) mice to explore the roles of TLR4 in the pathogenesis of biliary injury within a resistant strain mice by *C. sinensis*. Surprisingly, different from the roles of TLR4 in a susceptible mouse model demonstrated by our previous study, in the present study, we found that TLR4 *^def^* mice showed an aggravation of the biliary injuries and peribiliary fibrosis caused by *C. sinensis*, which was associated with the increased type 2 immune responses in *C. sinensis*-resistant mice.

## Materials and Methods

### Ethics

Animal care and all experimental perform in this study were strictly conformed to the guidelines of the National Laboratory Animal Center. The main procedures and protocol were reviewed and approved by the Animal Care and Use Committee of Xuzhou Medical University License (2017-SK-05).

### Mice

Six- to 8-week-old TLR4*^wild^* mice (C57BL/10JNju) and TLR4*^def^* mice (C57BL/10ScN, TLR4*^def^*) were purchased from the Model Animal Research Center of Nanjing University and maintained under specific non-pathogenic conditions in the model animal research center of Xuzhou Medical University (Xuzhou, Jiangsu, China). The mice were housed in an air-conditioned room at 24°C with a 12-h dark/light cycle and permitted free access to food and water.

### Parasites Infection

Metacercariae of *C. sinensis* from *Pseudorasbora parva* were collected by digestion with a pepsin-HCl (0.6%) artificial gastric juice (Yan et al., 2015). In each infected group, 45 metacercariae were intragastrically administrated to the individual, and the irrigating solution was observed under the microscope to ensure that all the metacercariae were completely intragastrically administrated; the mice of the non-infected received the same volume of normal saline. On day 28th post-infection all the mice were sacrificed and the serum and liver tissues were collected for further study.

### Liver Function Test

The activities of alanine aminotransferase (ALT), aspartate aminotransferase (AST), total bilirubin (TBIL), alkaline phosphatase (ALP), total bile acid (TBA) were assayed in the Department of Laboratory Medicine, Affiliated Hospital of Xuzhou Medical University, China to indicate for hepatocellular and biliary injuries in infected mice and uninfected mice.

### Hematoxylin and Eosin Staining

Partial liver tissue (about 10 mm × 10 mm × 1 mm) was immersed in 4% paraformaldehyde for 48 h. The embedded tissue wax blocks were serially sectioned at 4 μm for hematoxylin and eosin (H&E) staining according to the manufacturer’s instructions (Jiangsu Beyotime biotechnology research institute, China). After sealing the slides with neutral adhesive, the pathological changes of stained histological sections were observed by microscope (Olympus, Japan).

### Masson’s Trichrome Staining

Four percent paraformaldehyde was used to fix the liver tissue from each strain of mice, then the liver was embedded in paraffin. Three- to 4-μm thick sections were prepared and stained with Masson’s trichrome according to the manufacturer’s instructions (Jiancheng, Nanjing, Jiangsu, China). The sections were observed under the microscope and digitized using an imaging system (Olympus, Japan). Five high-power visual fields (×400 magnifications, Olympus, Japan) were randomly selected from the staining sections of each mouse, and Image Pro Plus6.0 software was used to calculate the Integral optical density (IOD) of fibrous tissue. A higher IOD value means stronger positive expression.

### Immunohistochemistry Staining

Three- to 4-μm serial thick sections of embedded tissue from each mouse were used for immunohistochemical staining of cytokeratin 19 (CK-19), ki67, alpha-smooth muscle Actin (α-SMA). The liver tissue was deparaffinized, hydrated, and heated in citric acid buffer at 95°C for 10 min and then blocked with 5% BSA for 30 min. The slides were then incubated overnight with primary Anti-Cytokeratin 19(1:500, ab52625, Abcam, Cambridge, US), ki67 (1:400, ab15580, Abcam, Cambridge, US), alpha-smooth muscle Actin (α-SMA) (1:400, ab124964, Abcam, Cambridge, US). After washing with PBS, DAB (1:200, ZSGB–BIO, Beijing, China) as an enzyme-substrate was added. Five high-power fields (×100 magnifications, Olympus, Japan) were randomly selected from each mouse staining section. CK19, ki67, α-SMA positive expression was calculated by the software of Image J (NIH, Bethesda, USA).

### Flow Cytometry Analysis

Intrahepatic leukocytes were obtained as descript elsewhere with minor modification (Blom et al., 2009). Partial liver tissue from each mouse was minced and grinded gently through a 40 µm-gauge nylon strainer using a sterile syringe plunger, the preparation was then centrifuged at 1,500 rpm for 10 min, the cells were resuspended using 4 ml 40% Percoll (GE Healthcare, catalog number: 17-0891-01) and then transferred into a new tube containing 5 ml 80% Percoll slowly. Subsequently, the gradient solution was centrifuged at 2,500 rpm for 25 min, the leukocytes were obtained from the interphase between 40% Percoll and 80% Percoll. Red blood cells in cell pellets were removed using ACK Lysis Buffer (BD Biosciences, catalog number: 555899, USA). Approximate 10^7^ cells were subjected to cell surface phenotyping by flow cytometry. A panel of antibodies used as markers for detection as follows: anti-CD45 was labeled with PE, anti-CD11b was labeled with PerCP-Cy5.5, anti-CD86 was labeled with PE-Cy™7, anti-CD206 was labeled with Alexa Fluor 647 (all antibodies from BD PharMingen, San Diego, CA, USA). The cells were incubated with antibodies directed toward various cell surface antigens and in the dark on ice for 30 min, following the manufacturer’s recommendations (BD PharMingen, San Diego, CA, USA). Subsequently, the cells were washed by centrifugation at 1500 rpm for 10 min, resuspended in 400 µl FACS buffer, and analyzed utilizing FACSCantoII flow cytometer (Becton Dickinson, San Jose, CA, USA).

### Enzyme-Linked Immunosorbent Assay

Serum and mouse liver homogenate from each mouse was immediately subjected to evaluate the concentration of IgE, IL-4, IL-6, IL-10, IL-13, MCP-1, and TNF-α by commercial Enzyme-linked Immunosorbent Assay (ELISA) Kits (Thermo Scientific, US). All procedures were performed according to the instructions provided by the kit. Concentrations of cytokine in the sera were calculated using standard curves as references.

### Quantitative Real-Time Fluorescence PCR

Total RNA was isolated from the liver by the use of Trizol (Vazyme, Nanjing, China). According to the manufacturer’s instruction, RNA was reverse transcribed into cDNA by use of the FastQuant RT First Kit With gDNase (TIANGEN, Beijing, China). Primers for mouse: *NOS2* F: GGCAGCCTCTTGTCTTTGACC; R: GGGAATCTTGGAGCGAGTTGT; *Tnfa* F: CTCCTCCACTTGGTGGTTTGT; R: GGTGCCTATGTCTCAGCCTCT; *Arg1* F: GTCAGTCCCTGGCTTATGGTT; R: CAGCAGAGGAGGTGAAGAGTA; *Ym1* F: GGATGGCTACACTGGAGAAA; R: AGAAGGGTCACTCAGGATAA; *Fizzl* F: CCCTCCACTGTAACGAAG; R: GTGGTCCAGTCAACGAGTAA; *ILIb* F: TGTGTTTTCCTCCTTGCCTCTGAT; R: TGCTGCCTAATGTCCCCTTGAAT; *IL6* F: TCACAGAAGGAGTGGCTAAGGACC; R: ACGCACTAGGTTTGCCGAGTAGAT; *Tgfb* F: CCACCTGCAAGACCATCGAC; R: CTGGCGAGCCTTAGTTTGGAC;*Col-1a* F: CAGGGTATTGCTGGACAACGTG; R: GGACCTTGTTTGCCAGGTTCA

The real-time quantitative PCR was performed using a Roche 480 detection system using SYBR Green PCR master mix solution (Roche Diagnostics Ltd, Shanghai, China). Fold changes of each gene to that of control mice were calculated using the formula of 2^−ΔΔCt^, which was normalized by β-actin. The speciﬁcity of each PCR was monitored by Roche 480 detection system with melt-curve analysis.

### Detection of Hydroxyproline Contents

The hydroxyproline content of the mouse liver were assayed to indicate for collagen deposition of liver in the infected mice and uninfected mice using a commercial kit (Nanjing JianCheng Technology Co. LTD, China). The assay was performed according to the instructions provided in the kit.

#### Statistical Analysis

All values were expressed as mean ± SEM. The data were analyzed by SPSS19.0 software (SPSS Inc, Chicago, IL, USA). The student *t-*test was used for comparison between the two groups. One-way ANOVA with Tukey’s *post hoc* test was used for comparison for more than two groups. If the test level was α=0.05, the difference was statistically significant when *P*< 0.05.

## Results

### TLR4*^def^* Accelerate Hepatic Damages in *C. sinensis*-Infected Mice

**Figure 1:**
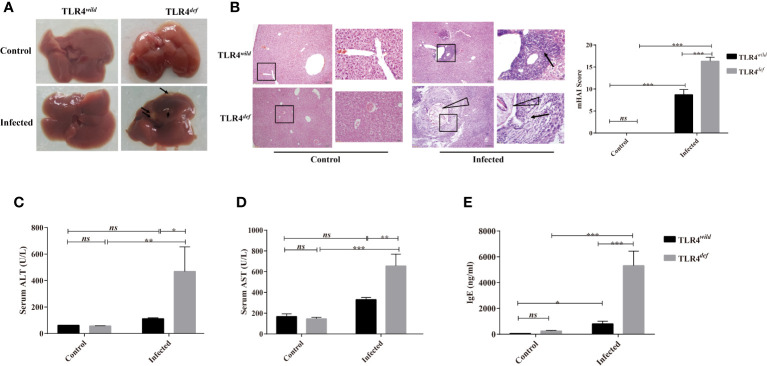
Toll-like receptor 4 (TLR4) deficiency aggravated liver injuries in C57BL/10 mice infected by *Clonorchis sinensis*. **(A)** Gross lesions in liver tissue of TLR4*^wild^* and TLR4*^def^* mice infected with *C. sinensis*. The arrow indicates the lesion nodule. **(B)** Histological changes of liver in TLR4*^wild^* and TLR4*^def^* mice infected with *C. sinensis* under 100× and 400× microscope were indicated by HE staining and liver histopathological changes were assessed by the mHAI score. An arrow indicates the infiltrated immune cells; triangle indicates worm bodies in the bile ducts. **(C)** The activities of alanine aminotransferase (ALT) and **(D)** aspartate aminotransferase (AST) were assessed in the sera of TLR4*^wild^* and TLR4*^def^* mice infected with *C. sinensis*. **(E)** The secretion of IgE in sera of TLR4*^wild^* and TLR4*^def^* mice infected with *C. sinensis* were determined using ELISA. The values were expressed as mean ± SEM. Compared with indicated groups, *P < 0.05, **P < 0.01, ***P < 0.001.

There were no obvious changes in the liver between TLR4*^wild^* and TLR4*^def^* mice without infection of *C. sinensis* (**Figure 1A**). However, following *C. sinensis* infection, gross changes of the liver in TLR4 wild-type infected mice were scarce. In contrast, the liver of TLR4*^def^* mice infected by *C. sinensis* had a dark color, tough texture, and uneven edges. Multiple white sesame-sized nodules were visible and most of them were lesions distributed on the left lobe of the liver (**Figure 1A**). Histological changes of the liver collected from each mouse were observed by H&E staining (**Figure 1B**). HE staining showed that the hepatocytes in the normal group were arranged neatly, the hepatic lobule structure was complete, and there was no inflammatory cell infiltration in the portal area, bile duct expansion, or hyperplasia (**Figure 1B**). Following *C. sinensis* infection, the histological analysis of the liver of wild-type infected mice showed an infiltration of inflammatory cells, with thickened bile duct hyperplasia and a small amount of fibrous tissue hyperplasia (**Figure 1B**). However, the overall hepatic lobule structure was orderly, the damaged area was relatively limited, and the hepatocytes were orderly arranged. Worm bodies in the bile ducts were not observed within the staining of liver tissues. However, TLR4*^def^* infected mice had very serious liver damage: a large number of inflammatory cells were infiltrated around the bile duct, the bile duct epithelium was disrupted, the hepatic lobule structure was damaged, and the liver cells were arranged in disorder (**Figure 1B**, as indicated by arrows). Liver inflammation was increased as indicated by the mHAI score (**Figure 1B**, *P*<0.05). For the bile duct, it could be seen that the bile duct had a complete structure with single-layer epithelium in the non-infected groups of mice. However, when the mice were infected by *C. sinensis*, TLR4*^wild^* mice showed an irregular bile duct and mild proliferation of BECs accompanied by only a few inflammatory cells infiltrated. Compared with TLR4*^wild^* mice infected by *C. sinensis*, TLR4*^def^* infected mice showed that the bile duct was more severe and irregular in shape, where mature worms bodies could be observed (indicated as triangle, **Figure 1B**). Furthermore, we evaluated hepatic damages as indicated by the activities of ALT and AST. It was found that the levels of ALT and AST in TLR4*^def^* infected mice were significantly higher than those of TLR4*^def^* uninfected mice. Meanwhile, compared with TLR4*^wild^* infection group, the activities of ALT and AST were significantly increased in TLR4*^def^* infection group (**Figures 1C, D**, *P*<0.05).

It has been reported that the increased production of IgE during the worms infection is related to tissue damages (Hamid et al., 2015). The data showed that the serum IgE secretion by TLR4*^wild^* infected mice was slightly higher than that of TLR4*^wild^* non-infected mice (**Figure 1E**, *P*<0.05). However, the serum IgE secretion in TLR4*^def^* infected mice was higher than that of the non-infected group, as well as that of TLR4*^wild^* infected mice (**Figure 1E**, *P*<0.001).

### TLR4*^def^* Aggravates Liver Fibrosis in Mice Infected by *C. sinensis*

**Figure 2:**
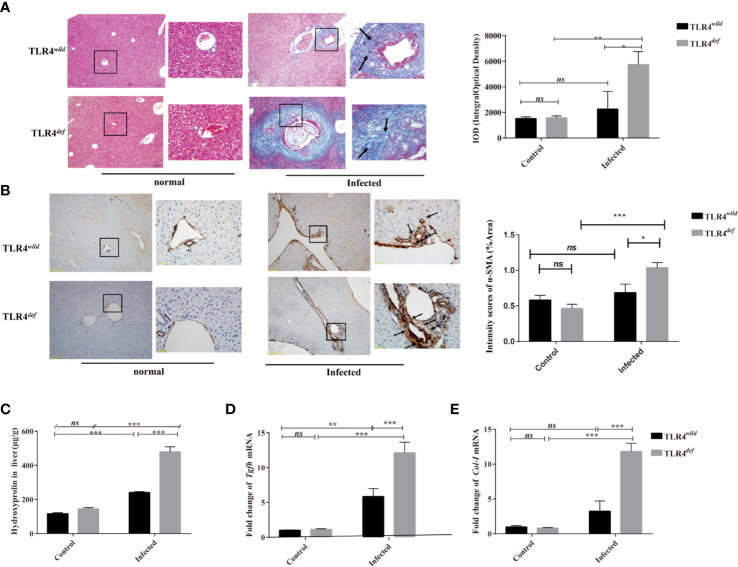
TLR4*^def^* led to an increased *Clonorchis sinensis*-caused pathogen-associated liver fibrosis in C57BL/10 mice. **(A)** Masson’s staining showed collagen depositions of the liver in TLR4*^wild^* and TLR4*^def^* mice infected with *C. sinensis* under 100× and 400× microscope. The integral optical density (IOD) of collagen fibers indicated by Masson’s trichrome staining was digitized and quantitated in the liver of non-infected and infected mice by on Image-Pro Plus software. **(B)** The expression of α-SMA of the liver in mice infected with *C. sinensis* under 100× and 400× microscope. Arrow indicates the positive cells. The positive areas in the liver of non-infected and infected mice were quantitated by Image J. **(C)** Detection of hydroxyproline in mice liver after *C. sinensis* infection. **(D, E)** Fold expression of fibrotic related molecules in *Tgfb* **(D)**, *Col1a* **(E)** in the liver of mice infected with *C. sinensis*. The values were expressed as mean ± SEM. Compared with indicated groups, *P < 0.05, **P < 0.01, ***P < 0.001.

To observe and evaluate the liver fibrosis caused by *C. sinensis*, we used Masson’s trichrome staining and the expression of α-SMA was used for assessment of the degree of liver fibrosis. Masson staining showed that no obvious collagen deposition was observed in the non-infected groups. However, following *C. sinensis* infection, the hyperplasia of fibrous tissue in TLR4*^wild^* infected mice was mainly deposited around the bile duct, although the overall damage was relatively limited. In contrast, TLR4*^def^* infected mice had a large amount of fibrous tissue hyperplasia around the diseased bile duct (indicated as arrows, **Figure 2A**). The depositions of collagen in TLR4*^def^* infected mice were increased compared with the non-infected group (**Figure 2A**, *P*<0.05), whereas the mice in the TLR4*^def^* infected group had more fibrosis than those in the TLR4*^wild^* infected group (**Figure 2A**, *P*<0.05). IHC showed that the expression of α-SMA was slightly increased after *C. sinensis*-infected TLR4*^wild^*mice, compared with non-infected mice (**Figure 2B**). However, the expression of α-SMA was significantly increased in *C. sinensis*-infected TLR4*^def^* mice and there were statistical differences in the expression of α-SMA between TLR4*^wild^* and TLR4*^def^* mice that were infected with the same amount of *C. sinensis* (positive areas indicated as arrows, **Figure 2B**, *P*<0.05). Furthermore, the concentrations of hydroxyproline in TLR4*^def^* infected mice was increased compared with the non-infected group (**Figure 2C**, *P*<0.001), and the mice in the TLR4*^def^* infected group had more fibrosis than those in the TLR4*^wild^* infected group (**Figure 2C**, *P*<0.001). We further detected the relative hepatic expression of *Tgfb* and *Col1a* transcripts in the livers of TLR4*^wild^* as well as TLR4*^def^* mice infected by *C. sinensis* by qRT-PCR. It was found that the expression of *Tgfb and Col1a* mRNA transcripts in the liver of TLR4*^def^* mice were significantly increased compared with TLR4*^wild^* mice after *C. sinensis* infection (**Figures 2D, E**, *P*<0.001). Taken together, our data suggested that TLR4 deficiency aggravated hepatic fibrosis in C57BL/10 mice infected by *C. sinensis*.

### TLR4*^def^* Exacerbates Bile Duct Hyperplasia and Biliary Injuries Caused by *C. sinensis*

**Figure 3:**
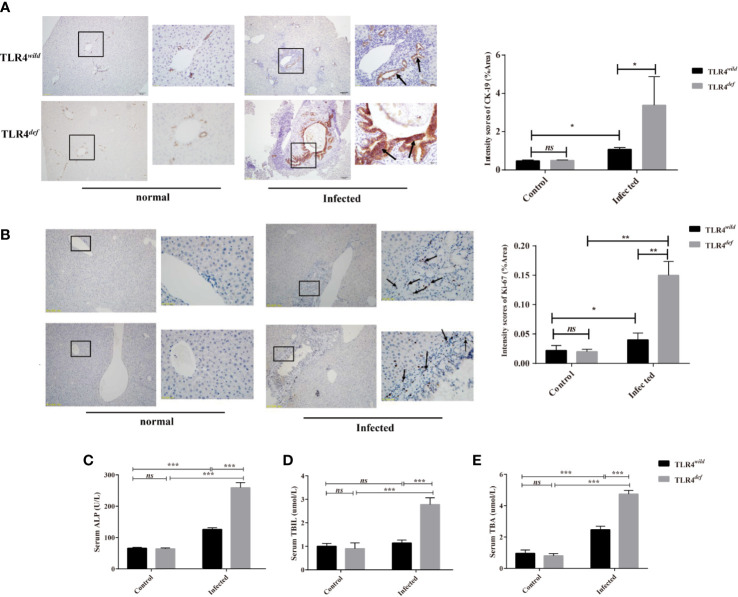
TLR4*^def^* promoted bile duct hyperplasia and biliary injuries in a resistant mouse strain of *Clonorchis sinensis* infection. **(A, B)** Hyperplasia of intrahepatic bile duct epithelial cells in mice with *C. sinensis* was indicated by immunohistochemical staining of CK19 **(A)** and ki67 **(B)**, an arrow indicates the positive cells. **(C–E)** The activities of ALP **(C)**, TBIL **(D)**, and TBA **(E)** in the sera of TLR4*^wild^* and TLR4*^def^* mice were assessed for biliary injuries. The values were expressed as mean ± SEM. Compared with indicated groups, *P < 0.05, **P < 0.01, ***P < 0.001.

In the present study, we detected CK19 and ki67 as indicators of the hyperplasia of biliary epithelium cells (BECs) and the morphology of the bile duct by immunohistochemistry. For semi-quantitative analysis for CK19 and Ki67 expression, compared with non-infection and TLR4*^wild^*-infected mice, TLR4*^def^* infected mice showed higher expression of CK19 and Ki67 (as indicated by arrow), suggesting that TLR4*^def^* infected mice had more bile duct hyperplasia than TLR4*^wild^* infected mice (**Figure 3A** for CK19, and **Figure 3B** for Ki67, *P*<0.01). In addition, we also detected the serum activities of ALP, TBIL, and TBA which also indicated biliary injuries. The data showed that the levels of these indicators in TLR4*^def^* mice infected by *C. sinensis* were significantly increased, compared with those in TLR4*^wild^* mice when they were infected with the same dose of *C. sinensis* (**Figures 3C–E**, *P*<0.001). Taken together, our data indicated that TLR4 deficiency in mice with the C57BL/10 genetic background exacerbated hyperplasia and injuries of cholangiocytes caused by *C. sinensis*.

### TLR4*^def^* Promotes the Activation of M2-Like Macrophages in *C. sinensis*-Infected Mice

**Figure 4:**
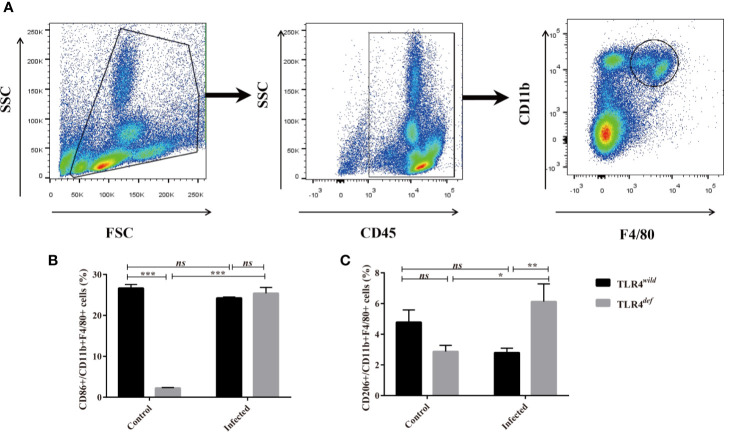
TLR4*^def^* mice infected with *Clonorchis sinensis* induced an predominant M2-like macrophage. **(A)** The strategies of gating hepatic macrophages. Hepatic leukocytes were isolated from TLR4*^def^* and TLR4*^wild^* mice, these cells were gated by CD45, and further gating for CD11b^+^F4/80^+^ cells using flow cytometer. **(B)** The percentages of CD86^+^cells in CD11b^+^F4/80^+^ cells from all the mice were calculated. **(C)** The percentages of CD206^+^cells in CD11b^+^F4/80^+^ cells from all the mice were calculated. The values were expressed as mean ± SEM. Compared with indicated groups, *P < 0.05, **P < 0.01, ***P < 0.001.

To investigate the possible mechanisms that may contribute to the deteriorative clonorchiasis in TLR4*^def^* mice, we detected hepatic M1/M2-like macrophages in mice after *C. sinensis* infection. It was found that there were no significantly increased in percentages of CD86^+^ in CD11b^+^F4/80^+^ macrophages (M1-like macrophage) in TLR4*^def^* mice, compared with TLR4*^wild^* mice when they were infected with the same amounts of *C. sinensis* (**Figure 4B**, *P*>0.05), but the proportion of CD206^+^ in CD11b^+^F4/80^+^ macrophages (M2-like macrophage) in *C. sinensis*-infected TLR4*^def^* was significantly higher than that in TLR4*^wild^* mice infected by *C. sinensis* (**Figure 4C**, *P*<0.05).

### Loss of TLR4 Increases Type 2 Immune Responses but Decreases Type 1 Responses in *C. sinensis*-Infected Mice

**Figure 5:**
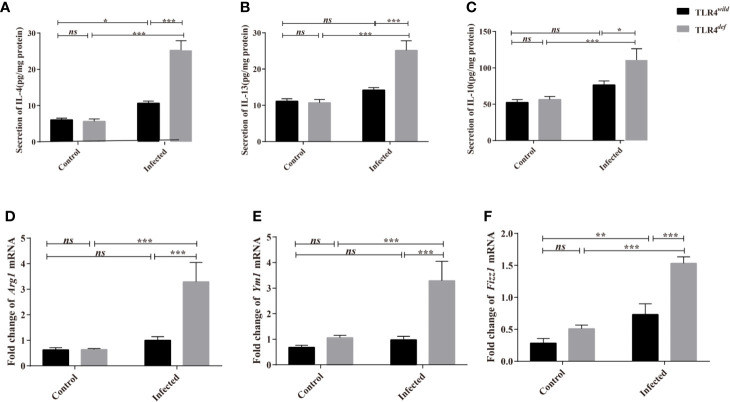
TLR4*^def^* enhanced type 2 immune response in C57BL/10 mice infected by *Clonorchis sinensis*. **(A~C)** The hepatic concentrations of IL-4 **(A)**, IL-13 **(B)**, and IL-10 **(C)** in the TLR4*^wild^* and TLR4*^def^* mice infected with *C. sinensis* were detected using ELISA kits. **(D~F)** The hepatic levels of *Arg1* **(D)**, *Ym1* **(E)**, *Fizz1* **(F)** were determined by qPCR. The values were expressed as mean ± SEM. Compared with indicated groups, *P < 0.05, **P < 0.01, ***P < 0.001.

We next detected the levels of type 1 immune responses and type 2 immune responses of TLR4*^wild^* and TLR4*^def^* mice after *C. sinensis* infection. The data showed that there were significantly increases in IL-4, IL-10, and IL-13 cytokines in TLR4*^def^* mice, compared with TLR4*^wild^* mice when they were infected by the same amounts of *C. sinensis* (**Figures 5A–C**, *P*<0.05). Furthermore, we detected another type 2 immune molecules such as *Arg1*, *Ym1* and *Fizz1* using qPCR, it was found that the relative expression of *Arg1*, *Ym1* and *Fizz1* in the livers of *C. sinensis*-infected TLR4*^def^* mice were significantly higher than those in mice in *C. sinensis*-infected TLR4*^wild^* mice (**Figures 5D–F**, *P*<0.001).

**Figure 6:**
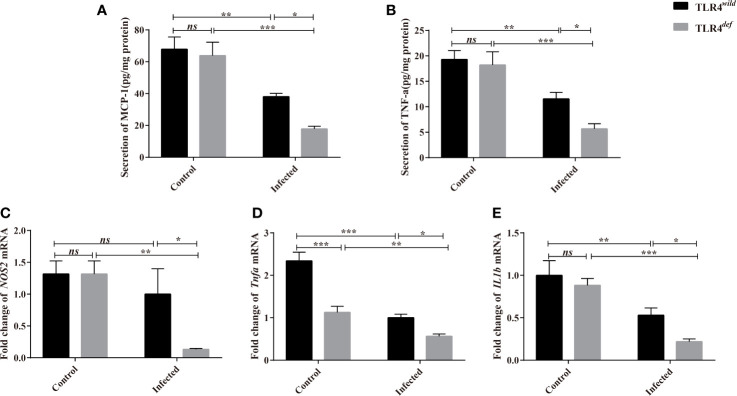
TLR4*^def^* decreased type 1 immune responses in C57BL/10 mice infected by *Clonorchis sinensis*. **(A, B)** The hepatic concentrations of MCP-1 **(A)** and TNF-e **(B)** in the TLR4*^wild^* and TLR4*^def^* mice infected with *C. sinensis* were detected using ELISA kits. **(C, D)** Fold changes of type 1 immunity-related molecules in *NOS2* **(C)**, *Tnfa* **(D)**, *IL1b* **(F)** in the liver of mice infected with *C. sinensis* from each group were determined by qPCR. The values were expressed as mean ± SEM. Compared with indicated groups, *P < 0.05, **P < 0.01, ***P < 0.01.

For type 1 immune responses, it was found that the secretion of MCP-1 and TNF-α in the liver of *C. sinensis*-infected TLR4*^def^* was significantly lower than that in TLR4*^wild^* mice infected by *C. sinensis* (**Figures 6A, B**, *P*<0.05). And the qPCR data showed that the transcripts of *NOS2, Tnfa*, and *IL1b* in the liver of TLR4*^def^* mice were significantly depressed, compared with TLR4*^wild^* mice after *C. sinensis* infection (**Figures 6C–F**, *P*<0.05). These data together suggested that TLR4*^def^* mice induced an augment of type 2 immune responses and decrease type 1 response, which might result in the exacerbation of biliary injuries and peribiliary fibrosis caused by *C. sinensis* in a resistant mice strain.

## Discussion

Mice are widely used to mimic pathogenesis in human-caused by *C. sinensis* since they have gall-bladder and the genetic background is clear enough and many reagents are commercially available. However, other and our previous studies demonstrated that mice with different genetic backgrounds showed distinct susceptibility to *C. sinensis*. For example, liver damage of C3H/HeN (with the normal function of TLR4), BALB/c and FVB mice infected mice were characterized by massive hyperplasia and disordered arrangement of bile duct epithelial cells, as well as deposition of extracellular matrix (Yoon et al., 2001; Zhang et al., 2017). Furthermore, adult worm bodies can be found in the bile ducts of these strains of mice. In contrast, Uddin et al. found that C57BL/6 mice had the lowest worm recovery at 4 weeks after infection and the worms were less developed, which induced mild histological changes in the liver (Uddin et al., 2012). The lesions of C57BL/10 or C57BL/6 infected mice were very limited, with only a small amount of inflammatory cell infiltration in the lesions when they were subjected to the same amount of *C. sinensis* (Zhang et al., 2017). In addition, mild fibrosis lesions indicating by Masson staining and hydroxyproline assay were also found in *C. sinensis*-infected C57BL/10 and C57BL/6, which further suggested that the susceptibility of different strains of mice to *C. sinensis* was different (Robey et al., 2015). The mechanisms accounting for these may be very complex and largely unknown. In our present study, in contrast with TLR4 wild-type mice, we found that the loss of TLR4 in C57BL/10 mice showed exacerbated biliary injuries and peribiliary fibrosis caused by *C. sinensis*, which may be associated with an increase of type 2 immune responses due to TLR4 deficiency. There were some similarities or disparities of our data from TLR4 deficiency mice with patients infected by *C. sinensis*, for example, TLR4 deficiency mice showed relatively high levels of liver function indexes (such as ALP, ALT, AST, TBIL, and TBA, etc), IgE antibodies subclass as well as type 2 immune responses, which were also elevated in human infected by *C. sinensis* (Cai et al., 2004; Han et al., 2015); however, we did not find an increase of type 1 immune responses in TLR4*^def^* mice infected by *C. sinensis* whereas human with *C. sinensis* infection showed high levels pro-inflammatory cytokines such as TNF-α (Yuan et al., 2013).

A high level of IgE production is a hallmark of infection by helminth. It is believed that IgE plays a central role in protective immunity against worm infection although the mechanism underlying it remains unclear (Cooper et al., 2008). It also suggested that IgE induced the activation of mast cells to recruit immune effector cells including Th2 cells, M2 macrophage, and eosinophils and trigger type II cytokines (i.e. IL-4, IL-5, and IL-13), leading to cell hyperplasia and tissue injuries due to the hypersensitivity reactions (Gurish et al., 2004; Kondo et al., 2008). Interestingly, treatment with monoclonal IgE antibody (omalizumab, Xolair) alleviates allergic reactions and airway damages in asthma by blockade of IgE binding with Fc εRI receptor on the mast cells and other effector cells (Corren et al., 2017). In our present study, we found that the relative higher IgE production in TLR4 deficiency mice was induced by *C. sinensis*, compared with TLR4 wild type mice, suggesting that IgE may be involved in the pathogenesis caused by *C. sinensis* in addition to expelling worms.

TLR4, as an ancient and conservative pattern recognition receptor, plays an important role in the invasion, colonization, pathogenesis, and elimination of pathogens, and may display multiple effects at different stages of disease development (Shah et al., 2012; Cheng et al., 2015). Recently, it has been reported that the TLR4-mediated NF-κB signaling pathway which can cross-talk with the TGF-β/samds signaling pathway plays a very important role in the pathogenesis of hepatic fibrosis and cholestatic injuries (Seki et al., 2007). Our previous study has used C3H/HeN (TLR4 wild) and C3H/HeJ (TLR4 mutation) strain mice to establish the *C. sinensis* infection model and found that the biliary fibrosis of TLR4 mutant mice (TLR4*^mut^*) was ameliorative, compared with wild-type mice, which suggested that TLR4 promotes liver fibrosis caused by *C. sinensis* (Yan et al., 2017). In contrast, in the present study, TLR4 deficiency in a resistant mouse strain following *C. sinensis* infection deteriorated the biliary injuries and biliary fibrosis, as indicated by CK19 immunohistochemical staining and Masson staining, respectively, which was consistent with previous studies (Yang et al., 2017; Zhang et al., 2017). Furthermore, we also found that the liver function was also damped to some extent as the activities of ALT were significantly increased in the TLR4 deficient mouse infected by *C. sinensis*, compared with TLR4 wild type with the same background. These data suggested that TLR4 might play contradictory roles in pathogenesis caused by *C. sinensis* due to the different genetic backgrounds of mice (Zhang et al., 2018).

Type 1 immune responses are characterized with CD4+Th1, classically activated macrophages (M1 macrophages), group 1 innate lymphoid cells (ILC1s), and cytotoxic T cells, which protect against intracellular microbes and enhance cell-mediated immunity by secretion of cytokines such as TNF-α, IL-6, INF-γ, and MCP-1 and secretion of NO (Annunziato et al., 2015); Type 2 immune responses are mediated by CD4+Th2 cells, group 2 ILCs, eosinophils, basophils, mast cells, M2 macrophages by producing the cytokines IL−4, IL−5, IL−9, IL−13, and the IgE antibody subclass, which suppress the development of type 1-driven inflammation and contribute to the pathogenesis of allergy and tissue fibrosis (Wynn, 2015). In the healthy animals, the type 1 and 2 immune responses of the host are finely regulated; however, their balance might determine the occurrence, development, and outcome of the disease. For example, during worm infection, Type 2 immune responses were overwhelmed in the chronic infection to protect from such large extra−cellular helminth parasites infection as well as facilitate tissue fibrosis (Wynn, 2015). In our previous study, compared with BALB/c and FVB mice, we found that C57BL/6 mice showed stronger resistance to *C. sinensis* infection and the worm body was not completely developed or retarded and even eliminated (Zhang et al., 2017). It was speculated that these differences might be closely related to the high level of type 2 immune responses in the liver of BALB/c and FVB mice (Zhang et al., 2017). The type 1 immune response was predominant in C57BL/6 mice. For example, macrophages of C57BL/6 mice can effectively eliminate the bacterial infection in the model of cecal ligation and perforated peritonitis by producing high levels of TNF-α, IL-12 and NO, and other types I immune response molecules (Uddin et al., 2012; Pine et al., 2018). However, BALB/c mice have a dominating type II immune response and could not eliminate bacterial infections effectively (Watanabe et al., 2004; Jovicic et al., 2015). Studies also have shown that type 2 immune response can promote the development and progression of liver fibrosis, thereby promoting the activation of HSCs by producing IL-10, Arg-1, Ym1, and Fizz1 (Liu et al., 2004) whereas pro-inflammatory mediators such as iNOS could inhibit the activation or induce the apoptosis of HSCs (Allen and Sutherland, 2014; Pine et al., 2018). In our present study, we focused on the hepatic macrophages which play a critical role in the cholestatic liver injuries including pathogenesis caused by *C. sinensis* (Kim et al., 2017; Guicciardi et al., 2018). To address this issue, we used CD86+CD11b+F4/80+ defining as an activation marker of M1 macrophage whereas CD206+CD11b+F4/80+ were used to assess M2 activation, we found that TLR4 deficiency in C57BL/10 increase M2 macrophage producing the molecules of *Arg-1, Ym1 and Fizz1* to possibly result in severe immunopathological damages (such as liver fibrosis), however, whether the biased type 2 immune responses induced by TLR4 deficiency may affect the development of the worms in the host or not, it remains to be further studied.

In conclusion, the present study found that biliary injuries and peribiliary fibrosis caused by *C. sinensis* had deteriorated when TLR4 was absent, suggesting that TLR4 might be involved in the resistance to *C. sinensis* in C57BL/10 mice. However, further studies on the mechanisms by which TLR4 deficiency in C57BL/10 mice induced pathogenesis of *C. sinensis* should be warranted.

## Data Availability Statement

The datasets generated for this study are available on request to the corresponding authors.

## Ethics Statement

The animal study was reviewed and approved by Animal Care and Use Committee of Xuzhou Medical University.

## Author Contributions

CY and K-YZ conceived and designed the experiments. CY, JW, and NX performed the majority of experiments. JL, Q-YZ, H-MY, X-DC, J-XL, XD, SK, QY, B-BZ, J-XC, and R-XT contributed to the acquisition of data. JW and CY wrote the paper. All authors contributed to the article and approved the submitted version.

## Funding

This study was supported by National Natural Science Foundation of China (Grant Nos: 81572019 to K-YZ and 81702027 to QY), Natural Science Foundation of Jiangsu Province of China (Grant No. BK20171176 to CY and Grant No. BK20201011 for B-BZ), China Postdoctoral Science Foundation (Grant No. 2018M640525 to CY), Qian Lan Project of Jiangsu Province (to CY), Jiangsu Planned Projects for Postdoctoral Research Funds (No. 2018K053B to CY), the starting grants for young scientist of Xuzhou Medical University (No. D2019040 to B-BZ), Priority Academic Program Development of Jiangsu Higher Education Institutions of China (Grant No. 1506 to K-YZ) and Graduate research project of Jiangsu Province (Grant No. KYCX18-2172 to JW). The funders had no role in study design, data collection and analysis, decision to publish, or preparation of the manuscript.

## Conflict of Interest

The authors declare that the research was conducted in the absence of any commercial or financial relationships that could be construed as a potential conflict of interest.
